# DNA copy number profiles of gastric cancer precursor lesions

**DOI:** 10.1186/1471-2164-8-345

**Published:** 2007-10-01

**Authors:** Tineke E Buffart, Beatriz Carvalho, Thomas Mons, Rui M Reis, Cátia Moutinho, Paula Silva, Nicole CT van Grieken, Michael Vieth, Manfred Stolte, Cornelis JH van de Velde, Evelin Schrock, Anja Matthaei, Bauke Ylstra, Fátima Carneiro, Gerrit A Meijer

**Affiliations:** 1 Department of Pathology, VU University Medical Center, Amsterdam, The Netherlands; 2 Institute of Pathology and Molecular Immunology of University of Porto – IPATIMUP, Porto, Portugal; 3 Life and Health Sciences Research Institute (ICVS), Health Sciences School, University of Minho, Portugal; 4 Institute of Pathology, Klinikum Bayreuth, Bayreuth, Germany; 5 Dept. Surgery, Leiden University Medical Center, Leiden, The Netherlands; 6 Institute of Clinical Genetics, University of Technology, Dresden, Dresden, Germany; 7 Faculty of Medicine, University of Porto and Hospital, S. Joao, Porto, Portugal

## Abstract

**Background:**

Chromosomal instability (CIN) is the most prevalent type of genomic instability in gastric tumours, but its role in malignant transformation of the gastric mucosa is still obscure. In the present study, we set out to study whether two morphologically distinct categories of gastric cancer precursor lesions, i.e. intestinal-type and pyloric gland adenomas, would carry different patterns of DNA copy number changes, possibly reflecting distinct genetic pathways of gastric carcinogenesis in these two adenoma types.

**Results:**

Using a 5K BAC array CGH platform, we showed that the most common aberrations shared by the 11 intestinal-type and 10 pyloric gland adenomas were gains of chromosomes 9 (29%), 11q (29%) and 20 (33%), and losses of chromosomes 13q (48%), 6(48%), 5(43%) and 10 (33%). The most frequent aberrations in intestinal-type gastric adenoma were gains on 11q, 9q and 8, and losses on chromosomes 5q, 6, 10 and 13, whereas in pyloric gland gastric adenomas these were gains on chromosome 20 and losses on 5q and 6. However, no significant differences were observed between the two adenoma types.

**Conclusion:**

The results suggest that gains on chromosomes 8, 9q, 11q and 20, and losses on chromosomes 5q, 6, 10 and 13, likely represent early events in gastric carcinogenesis. The phenotypical entities, intestinal-type and pyloric gland adenomas, however, do not differ significantly (P = 0.8) at the level of DNA copy number changes.

## Background

Gastric cancer is the second most frequent malignancy worldwide and the prognosis of this malignancy remains very poor [1]. Gastric cancer incidence and mortality rates differ between different countries within the European Union [2]. In the Netherlands it ranks fifth as a cause of cancer death, with approximately 2,200 new cases each year [3]. Surgery with curative intent is the treatment of choice in advanced cases of gastric cancer, whereas local endoscopic mucosectomy can be curative in early gastric cancer. Detection and removal of gastric neoplasias in an early or even premalignant state will contribute to reduce death due to gastric cancer. To achieve this goal, better tests for early detection of gastric cancer are needed, and an improved understanding of the biology of gastric cancer progression is crucial in this respect.

According to the Correa model, pathogenesis of intestinal-type gastric adenocarcinoma follows a pathway of chronic active gastritis due to *Helicobacter pylori *infection, leading to mucosal atrophy, intestinal metaplasia followed by intraepithelial neoplasia and finally invasive adenocarcinoma [4]. Genetic characterization of tissue samples in intraepithelial neoplasia stage would substantially contribute to our understanding of the molecular pathogenesis of gastric cancer. However, these lesions are only rarely detected, possibly due to rapid progression through this stage towards cancer, and are usually present only in parts of biopsy specimens, hampering genomic analysis of these lesions. Analysis of alternative precursor lesions could therefore, at least partly, be a substitute. Development of gastric cancer through an adenoma stage, although less common, is such alternative route. These adenomas are occasionally detected during gastroscopy and present as large lesions that histologically show intra-epithelial neoplasia, which makes them suitable for genomic analysis. Gastric adenomas have a direct malignant potential and account for approximately 20% of all epithelial polyps [5,6]. Gastric adenomas can have a classic tubular, tubulovillous, or villous morphology with a predominantly intestinal-type epithelium, but can also appear as pyloric gland adenomas [6]. Pyloric gland adenomas arise from deep mucoid glands in the stomach and are strongly positive for mucin 6 [7,8]. A substantial number of gastric adenomas already show progression to adenocarcinoma. On first diagnosis around 30–40% of all pyloric gland adenomas already show a focus of carcinoma [9,10]. For intestinal-type adenomas this number is lower and varies from 28,5% for villous adenomas and 29.4% for tubulovillous type adenomas to only 5.4% in the tubular adenomas [11]. Both adenocarcinomas, ex intestinal-type adenomas and ex pyloric gland adenomas, show glandular structures, in contrast to diffuse type gastric cancer.

A key feature in the pathogenesis of most gastric cancers, as in many other solid cancers, is chromosomal instability, resulting in gains and losses of parts or even whole chromosomes [12]. These chromosomal changes can be analyzed by comparative genomic hybridization (CGH). Several previous studies have detected genetic alterations in gastric adenomas using this technique, being gains on chromosome 7q, 8q, 13q, 20q, and losses on chromosome 4p, 5q, 9p 17p and 18q [13-16]. Although uncommon and only observed in adenomas with high grade intraepithelial neoplasia, high level amplifications have been detected on chromosomes 7q, 8p, 13q, 17q and 20q [13-16]. In gastric adenocarcinomas, consistently described chromosomal aberrations are gains on chromosome 3q, 7p, 7q, 8q, 13q, 17q and 20q and losses on chromosome 4q, 5q, 6q, 9p, 17p and 18q. High level amplifications have been repeatedly detected on 7q, 8p, 8q, 17q, 19q and 20q [14,17-23]. Yet, chromosomal aberrations, or DNA copy number changes, are not uniform in gastric cancer [24]. Subgroups with different patterns of DNA copy number alterations can be recognized, which have been shown to be associated with clinical outcome as well [25].

In the present study, we set out to study whether two morphologically distinct categories of gastric cancer precursor lesions, i.e. intestinal-type and pyloric gland adenomas, would carry different patterns of DNA copy number changes, possibly reflecting distinct genetic pathways of gastric carcinogenesis in the two adenoma types.

## Results

DNA copy number changes were observed in 10 out of 11 intestinal-type adenomas and 9 out of 10 pyloric gland adenomas. The mean number of chromosomal events, defined as gains and losses, per tumour was 6.0 (range 0–18), including 2.9 (range 0–14) gains and 3.0 (range 0–7) losses. In intestinal-type adenomas, the mean number of chromosomal events per tumour was 6.5 (range 0–18) of which 3.4 (range 0–14) gains and 3.1 (range 0–7) losses, and in the pyloric gland adenomas the mean numbers were 5.4 (range 0–9), 2.4 (range 0–7) and 3.0 (range 0–7) respectively.

In the intestinal-type gastric adenomas, the most common aberrations observed were gains on chromosomes 8, 9q and 11q, and losses on chromosomes 5q, 6, 10 and 13. In four adenomas (36.4%), gain of chromosome 11q23.3 was observed with a common region of overlap of 2.6 Mb. Gain of chromosome 9q was observed in four adenomas (36.4%) with a 12.6 Mb common region of overlap located on chromosome 9q33.1-q34.13. Gain of chromosome 8 was observed in three adenomas (31%), two of which adenomas showed gain of whole chromosome 8, and the third adenoma showed a gain of chromosome 8p-q22.3 with an additional 28.7 Mb gain on chromosome 8q24.11-qter. In addition, gains were observed on chromosomes 1, 3, 6p, 7, 11p, 12p, 13q, 16, 17, 19, 20 and 22q. No amplifications were seen in the intestinal-type adenomas.

Deletions on chromosome 13 were observed in seven intestinal-type adenomas (64%). Of these, five showed a 11.9 Mb deletion of chromosome 13q21.2-21.33 with an additional 7.7 Mb deletion on chromosome 13q31.1-31.3. The other two adenomas showed a 16.6 Mb deletion of 13q14.3-31. A deletion on chromosome 6 was observed in six adenomas (55%), with an overlapping region of 68.9 Mb located on 6cen-q22.1. A deletion of chromosome 5q was observed in four adenomas (36%) with a common region of overlap located on chromosome 5q22.1-q23.2. In addition, a deletion of whole chromosome 10 was observed in four adenomas (36%). Other losses observed in intestinal-type adenomas were located on chromosomes 8q, 9p, 10, 12q, 20q and 21. An overview of all DNA copy number aberrations of the intestinal-type adenomas is shown in Table 1.

**Table 1:** Overview of the DNA copy number changes in 11 intestinal-type adenomas

	**Chromosomal aberrations**		**Flanking clones**		
	
**Tumour ID**	**Gains**	**Losses**	**Segment size (Mb)**	**Start**	**End**
1	1p-p36.11		26.68	RP11-465B22	RP1-159A19
		5q13.2-q23.2	55.26	RP11-115I6	CTB-1054G2
	6p21.33-p21.1		13.78	RP11-346K8	RP11-227E22
		6p21.1-q16.1	52.05	RP11-89I17	RP3-393D12
	9q33.1-34.2		17.32	RP11-27I1	RP11-417A4
	11q23.3		4.80	RP11-4N9	RP11-730K11
		13q21.1-q31.3	39.63	RP11-200F15	RP11-62D23

2	1p-1p33		46.90	RP11-465B22	RP11-330M19
	6p21.33-p21.1		14.12	RP11-346K8	RP11-121G20
		6p21.1-q16.2	54.91	RP11-554O14	RP11-79G15
	8p-q22.3		105.67	GS1-77L23	RP11-200A13
	8q24.11-qter		28.65	RP11-278L8	RP5-1056B24
	9q33.1-q34.2		13.63	RP11-85O21	RP11-417A4
	11p11.2-q13.5		31.69	RP11-58K22	RP11-30J7
	11q23.3		2.62	RP11-4N9	RP11-62A14
	12q13.11-q14.1		10.57	RP11-493L12	RP11-571M6
		13q21.1-q21.33	18.24	RP11-200F15	RP11-335N6
		13q31.1-q31.3	12.49	RP11-533P8	RP11-62D23
	16p13.3-q21		57.26	RP11-243K18	RP11-405F3
		16q21-q22.1	5.97	RP11-105C20	RP11-298C15
	16q22.1-q24.3		22.46	RP11-63M22	CTC-240G10
	17		81.24	GS1-68F18	RP11-567O16
	19		61.01	CTB-1031C16	GS1-1129C9
	20q11.21-q11.23		5.09	RP3-324O17	RP5-977B1
	20q13.12-qter		19.60	RP1-138B7	CTB81F12

3	-	-			

4	6p21.1		3.32	RP11-79J5	RP11-121G20
		6p12.3-q22.1	76.38	RP11-79G12	RP11-59D10
	7		156.89	RP11-510K8	CTB-3K23
		8q22.3-q23.3	9.69	RP11-142M8	RP11-261F23
	9q33.1-q34.13		12.58	RP11-55P21	RP11-83N9
	11q23.3		3.04	RP11-4N9	RP11-8K10
		13q21.2-q21.33	17.05	RP11-240M20	RP11-77P3
		13q31.1-q31.3	11.68	RP11-400M8	RP11-100A3
	16q23.2-q24.3		8.92	RP11-303E16	RP4-597G12
	20p-q13.2		53.40	CTB-106I1	RP5-1162C3
	20q13.31-qter		8.06	RP5-1167H4	CTB-81F12
	22q		33.72	XX-P8708	CTB-99K24

5		12q24.31-qter	11.75	RP11-322N7	RP11-1K22

6	3		193.37	RP11-299N3	RP11-279P10
		6cen-q24.1	88.49	RP11-91E17	RP11-86O4
	7		156.09	RP11-510K8	RP11-518I12
	8		144.26	RP11-91J19	RP5-1118A7
		13q21.1-q21.33	11.86	RP11-640E11	RP11-452P23
		13q31.1-q31.3	9.62	RP11-400M8	RP11-306O1
		20q13.2-q13.31	1.41	RP11-212M6	RP4-586J11

7		5q21.1-qter	80.52	CTC-1564E20	RP11-281O15
		10	132.19	RP11-29A19	RP11-45A17
	13q21.33-31.1		8.76	RP11-209P2	RP11-470M1

8		5q22.1-q23.2	13.28	RP11-276O18	RP11-14L4
		6p12.3-q22.1	74.37	RP11-89l17	RP11-149M1
		9p21.1-pter	31.18	RP11-147I11	RP11-12K1
		10	133.18	RP11-10D13	RP11-45A17
		13q14.3-q31.3	39.71	RP11-211J11	RP11-306O1
	17		77.65	GS1-68F18	RP11-398J5
	19		63.31	CTC-546C11	CTD-3138B18
	20		60.87	RP4-686C3	RP4-591C20
	22q		31.25	XX-bac32	CTA-722E9

9		5q14.3-q23.2	33.06	RP11-302L17	RP11-14L4
		6p22.2-q22.3	8.44	RP11-91n3	RP11-88h24
		6p12.1-q24.1	88.89	RP11-7h16	RP11-368P1
	8		145.95	GS1-77L23	CTC-489D14
	9q33.1-qter		13.60	RP11-91G7	GS1-135I17
		10	133.18	RP11-10D13	RP11-45A17
	11q23.3		3.16	RP11-4N9	RP11-215D10
		13q14.3-qter	58.59	RP11-240M20	RP11-480K16
		20q13.2-q13.31	1.96	RP11-55E1	RP5-832E24
		21cen-q21.3	17.39	RP11-193B6	RP11-41N19

10		8q22.3-q23.3	12.93	RP11-142M8	RP11-143P23
		10	134.52	RP11-10D13	RP11-122K13
		13q21.1-q21.33	18.03	RP11-322F18	RP11-335N6
		13q31.1-q31.3	8.99	RP11-533P8	RP11-505P2

11	-	-			

The most frequent aberration observed in pyloric gland adenomas were gains on chromosome 20 and losses on chromosomes 5q and 6. Gains on chromosome 20 were seen in four adenomas (40%). Three adenomas showed a 9.8 Mb gain of chromosome 20q13.12-q13.33, and gain of whole chromosome 20 was observed in the other adenoma. In addition, gains were seen on chromosomes 1, 3q, 5q, 7, 9q, 11q, 12q, 13q, 15q, 17 and 22q. One pyloric gland adenoma showed amplifications, located on 12q13.2-q21.1 and 20q13.3-q13.33.

Five pyloric gland adenomas (50%) showed loss of chromosome 5q, two of which had lost a whole chromosome arm, while two adenomas showed a 22.4 Mb deletion of 5q11.2-q13.3 and one adenoma a 40.3 Mb deletion of 5q21.1-q31.2. Loss of chromosome 6 was observed in four pyloric gland adenomas (40%), three of which showed a complete loss of 6q and one adenoma showed a 51.2 Mb deletion of 6p21.1-q16.3. Other chromosomal losses were observed on chromosomes 1p, 2q, 4, 9p, 10, 12q 13q, 14q, 16, 18q, 20q, and 21. An overview of DNA copy number aberrations of the pyloric gland adenomas is shown in Table 2.

**Table 2:** Overview of the DNA copy number changes in 10 pyloric gland adenomas

	**Chromosomal aberrations**		**Flanking clones**		
	
**Tumour ID**	**Gains**	**Losses**	**Segment size (Mb)**	**Start**	**End**
12	1q21.3-q23.3		9.95	RP11-98D18	RP11-5K23
	1q42.13-q43		14.07	RP11-375H24	RP11-80B9
	3q		111.59	RP11-312H1	RP11-23M2
	5q35.1-q35.3		9.11	RP11-20O22	RP11-451H23
		6q	115.76	RP11-524H19	RP5-1086L22
	7		156.09	RP11-510K8	RP11-518I12
	17		77.48	RP11-4F24	RP11-313F15
	20		63.47	CTB-106I1	CTB-81F12

13	-	-			

14		4	191.13	CTC-963K6	RP11-45F23
		5q	128.59	CTD-2276O24	RP11-281O15
		14q	83.81	RP11-98N22	RP11-73M18
		16	89.71	RP11-344L6	RP4-597G12
	20q13.2-q13.33		10.84	RP4-724E16	CTB-81F12

15	9q33.2-q34.3		16.81	RP11-57K1	RP11-83N9
	11q23.2-q24.3		16.04	RP11-635F12	RP11-567M21
	12q14.3-q15		2.58	RP11-30I11	RP11-444B24
	20q13.31-q13.33		6.86	RP5-1153D9	RP5-963E22
	22q		32.53	XX-p8708	CTA-722E9

16	9q33.3-qter		13.57	RP11-85C21	GS1-135I17
		10p12.1-qter	110.28	RP11-379L21	RP11-45A17
	11q23.1-q24.3		17.72	RP11-107P10	RP11-567M21
		13q31.1-q32.1	10.84	RP11-661D17	RP11-40H10
		20q13.2-q13.31	1.96	RP11-55E1	RP4-586J11

17		1p34.3-pter	35.59	RP1-37J18	RP11-204L3
	1p33-qter		203.62	RP4-739H11	RP11-551G24
		2q31.1-qter	66.00	RP11-205B19	RP11-556H17
		5q21.1-q31.2	40.27	CTD-2068C11	RP11-515C16
	5q31.3-qter		39.06	CTD-2323H12	RP11-451H23
		6q	113.61	RP11-89D6	CTB-57H24
		10	134.52	RP11-10D13	RP11-122K13
		13q31.1-qter	36.14	RP11-388E20	RP11-245B11
		20q13.2-qter	11.24	RP11-15M15	RP5-1022E24

18		5q11.2-q21.2	51.24	CTC-1329H14	RP1-66P19
		6p12.1-q16.3	51.24	RP11-7H16	RP11-438N24
		9pter-q13	66.82	GS1-41L13	RP11-265B8
		10	133.04	RP11-10D13	RP11-45A17
		13q21.1-q21.33	18.39	RP11-240M20	RP11-335N6
		13q31.1-q31.3	12.45	RP11-551D9	RP11-100A3
		21cen-q21.3	17.39	RP11-193B6	RP11-41N19

19		1p32.3-p21.1	50.40	RP11-117D22	RP5-1108M17
		5q11.2-q13.3	24.64	RP4-592P18	CTD-2200O3
	13q12.11-q14.3		31.58	RP11-187L3	RP11-327P2
	15q12-q26.3		77.21	RP11-131I21	CTB-154P1
		18q21.1-q23	31.31	RP11-46D1	RP11-154H12
	22q13.2-qter		10.02	CTA-229A8	CTA-799F10

20		9p-q13	66.57	GS1-41L13	RP11-274B18
	12q13.2-q21.1 (amplification)		19.50	RP11-548L8	RP11-255I14
		12q21.2-qter	55.56	RP11-25J3	RP11-1K22
		18q21.31-q23	23.28	RP11-383D22	CTC-964M9
	20q13.13-q13.33 (amplification)		14.62	RP5-1041C10	RP5-1022E24

21	5p		43.15	CTD-2265D9	RP11-28I9
		5q	130.26	RP11-269M20	RP11-451H23
	6p		62.57	CTB-62I11	RP11-506N21
		6q	106.73	RP11-767J14	RP5-1086L22

**Figure 1:**
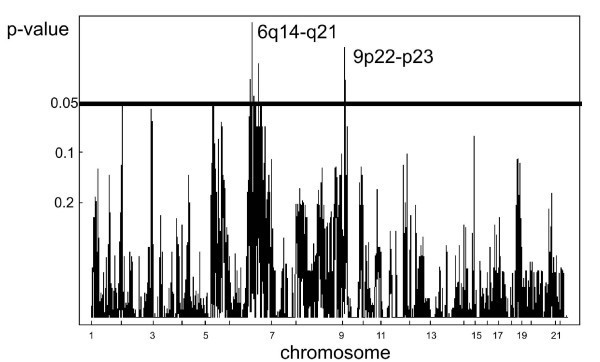
Comparison of DNA copy number alterations in intestinal and pyloric gland type gastric adenomas. A p-value (Y-axis) was calculated for every clone, based on a Wilcoxon test with ties, and plotted in chromosomal order from chromosome 1 to 22 (X-axis). Eight clones reached the level of significance (p < 0.05), but failed to maintain a significantly low false discovery rate after correction for multiple comparison.

The most common aberrations shared by both intestinal-type and pyloric gland adenomas were gain of chromosome 9q (29%), 11q (29%), and 20q (33%) and loss of chromosome 5 (43%), 6 (48%), 10 (33%) and 13q (48%). By comparing intestinal-type and pyloric gland adenomas, CGH Multiarray revealed eight clones to be significantly different, six of which were located at chromosome 6q14-q21 (p = 0.02 to 0.05) and two clones on chromosome 9p22-p23 (p = 0.02 and 0.04, respectively) (Figure 1). No genes located in the regions covered by these clones have been known to be involved in cancer related biological processes. Yet, CGH Multiarray Region, after correction for multiplicity, yielded a false discovery rate (FDR) of 1 for all these regions, indicating no significant differences between the two different types of adenomas at the chromosomal level. Unsupervised hierarchical cluster analysis yielded 2 clusters. No significant associations were found here (p = 0.8).

## Discussion

Given the heterogeneous phenotype of gastric cancer, the present study primarily aimed to compare copy number changes between intestinal-type adenomas and pyloric gland adenomas, in order to find leads towards genetic pathways involved in the pathogenesis of gastric cancer. Adenoma-to-carcinoma progression is observed in 30–40% of the pyloric gland adenomas and in approximately 5–30% of the intestinal-type adenomas (varying from about 5% in tubular adenomas to almost 30% for tubulovillous and villous adenomas) [9-11], indicating the direct malignant potential of these two adenoma types and making gastric adenomas a suitable model for detecting early events in gastric carcinogenesis.

Pyloric gland adenomas constitute a recently recognized entity [8,26]. To the best of our knowledge, this type of adenomas has never been analyzed by array CGH before. The mean number of events in this type of adenoma was 5.4 (0–9), with 2.4 (0–7) gains and 3 (0–7) losses. This is comparable with the mean number of aberrations in intestinal-type adenomas (6.5 (0–18), 3.4 (0–14) and 3.1 (0–7) respectively). In pyloric gland adenomas, frequent events were gain on chromosome 20 and losses on chromosomes 5q and 6, while intestinal-type adenomas mainly showed gain on chromosomes 8, 9q, and 11q, and losses on chromosomes 5q, 6, 10 and 13. In the present study, gain of chromosome 7 was less common than found previously [16]. Although these frequently altered regions differ between the two types of adenomas, hierarchical cluster analyses did not separate the groups. In addition, CGH Multiarray Region did not reveal any significant differences after correction for multiple comparisons. This lack of statistically significant differences could be due to the limited sample size combined with the fact that in general, adenomas show little chromosomal aberrations. On the other hand, it could simply be that these morphologically different entities do not differ in terms of chromosomal gains and losses. Finding no significant differences at the chromosomal level does not preclude other genetic and biological differences such as mutation or promoter methylation status of specific genes.

Aberrations already detected in adenomas may be early events in the stepwise process of accumulating changes which may cause progression of adenoma to carcinoma. As expected, the mean number of chromosomal events was lower in adenomas compared to the carcinomas [13,14,27]. Moreover, high level amplifications are uncommon in adenomas, while carcinomas frequently show high level amplifications [13,16].

The aberrations found in both intestinal-type and pyloric gland adenomas, such as losses on chromosome 5q, are also frequently detected in gastric carcinomas [15,19,28]. Previous CGH results showed a significantly higher number of chromosome 5q losses in intestinal-type carcinoma compared to diffuse type carcinoma [29]. Chromosome 6, also lost in both types of adenomas, frequently is deleted in gastric carcinomas as determined by LOH studies [30,31]. Moreover, chromosome 6q deletion has been reported to be involved in an early stage of gastric carcinogenesis, since chromosome 6q deletions are frequently detected in early gastric cancer and also in intestinal metaplasia [31,32]. Losses of chromosomes 10 and 13 have been previously observed in adenomas at lower frequencies. In gastric carcinomas, both gains and losses of chromosome 10 and 13 have been observed by previous CGH studies [15,19,21,33]. Chromosome 10 harbors the oncogene *FGFR2 *(10q26) and tumour suppressor genes *PTEN/MMAC1 *(10q23) and *DMBT1 *(10q25-q26), both involved in carcinogenesis, which could explain the observation of both gains and losses of chromosomes 10 in gastric carcinomas [34-36]. Indeed chromosome 13 harbors tumour suppressor genes such as *BRCA2 *(13q12.3) and retinoblastoma gene (*RB1*) (13q14). In contrast, gain of chromosome 13q has been correlated to colorectal adenoma-to-carcinoma progression, and amplification of chromosome 13 has been observed in gastric adenomas with severe intraepithelial neoplasia [14,37]. The precise role of chromosome 13 aberration in gastric cancer therefore remains to be resolved.

Most frequent copy number gains were observed on chromosomes 8, 9q, 11q and 20. Especially gains of chromosomes 8 and 20 are consistent with previous (array) CGH studies in both gastric adenomas and gastric carcinomas [13-16,19,25], implicating this as early events in tumourigenesis. Although gain of chromosome 11q has not been described as a frequent event in adenomas, in carcinomas gain or amplification on chromosome 11q is common [13-16]. In the present study gain of chromosome 11q was frequently observed in the adenomas, implying the malignant potential of these adenomas.

## Conclusion

These data indicate that gains on chromosomes 8, 9q, 11q and 20 and losses on chromosomes 5q, 6, 10 and 13 are early events in gastric carcinogenesis. Despite the phenotypical differences, intestinal-type and pyloric gland adenoma do not differ significantly at the level of DNA copy number changes.

## Methods

### Material

**Figure 2:**
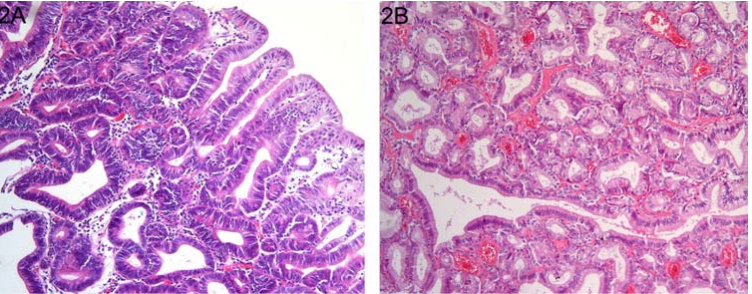
Haematoxilin and eosin staining (original magnification ×400) of intestinal-type (A) and pyloric gland (B) gastric adenomas. A. Intestinal-type adenoma of the stomach composed of irregularly arranged glands composed of intestinal-type epithelium with eosinophilic cytoplasm and enlarged nuclei. B. Pyloric gland adenoma of the stomach composed of densely back to back packed glands consisting of cells with pale cytoplasm and small round hyperchromatic nuclei.

Twenty-one paraffin-embedded gastric adenomas, 11 intestinal-type and 10 pyloric gland adenomas, were included in this study (Figure 2A and 2B). Tumour and patient data are given in Table 3. For each case, a tumour area consisting for at least 70% of tumour cells was demarcated on a 4 μm hematoxylin and eosin stained tissue section. Adjacent 10–15 serial tissue sections of 10 μm were stained with hematoxylin and the corresponding tumour area was microdissected using a surgical blade. A final 4μm "sandwich" section was made and stained with hemotoxylin and eosin, to compare with the first slide as a control. After deparaffinization, DNA was extracted by a column-based method (QIAamp DNA mini kit; Qiagen, Westburg, Leusden, NL) [38].

**Table 3:** Tumour and patient information

**Tumour ID**	**Adenoma type**	**Grade of dysplasia**	**Gender**	**Age**	**Tumour ID**	**Adenoma type**	**Grade of dysplasie**	**Gender**	**Age**
1	Intestinal	Moderate	Male	75	12	Pyloric gland	Moderate	Male	78
2	Intestinal	Moderate	Male	45	13	Pyloric gland	Mild	Male	50
3	Intestinal	Moderate	Male	80	14	Pyloric gland	Severe	Female	76
4	Intestinal	Moderate	Male	79	15	Pyloric gland	Moderate	Female	85
5	Intestinal	Moderate	Male	76	16	Pyloric gland	Moderate	Male	63
6	Intestinal	Moderate	Male	75	17	Pyloric gland	Mild	Female	86
7	Intestinal	Mild	Male	57	18	Pyloric gland	Moderate	Female	59
8	Intestinal	Moderate	Male	64	19	Pyloric gland	Moderate	Male	69
9	Intestinal	Mild	Male	63	20	Pyloric gland	Moderate	Female	78
10	Intestinal	Mild	Male	75	21	Pyloric gland	Moderate	Male	?
11	Intestinal	Moderate	Female	45					

Genomic DNA obtained from peripheral blood from ten normal individuals was pooled (either ten females or ten males, depending on the gender of the patient from which the adenoma was obtained) and used as control reference DNA.

Array CGH

Array CGH was performed essentially as described previously [39]. Briefly, 300 ng tumour and reference DNAs, sex-mismatch as experimental control, were labelled by random priming (Bioprime DNA Labelling System, Invitrogen, Breda, NL), each in a volume of 50μL. Non incorporated nucleotides were removed using ProbeQuant G-50 microcolumns (Amersham Biosciences). Cy3 labelled test genomic DNA and Cy5 labelled reference DNA were combined and co-precipitated with 100μg of human Cot-1 DNA (Invitrogen, Breda, NL) by adding 0.1 volume of 3 M sodium acetate (pH 5.2) and 2.5 volumes of ice-cold 100% ethanol. The precipitate was collected by centrifugation at 14,000 rpm for 30 minutes at 4°C, and dissolved in 130 μl hybridization mixture containing 50% formamide, 2 × SCC and 4% SDS. The hybridization solution was heated for 10 minutes at 73°C to denature the DNA, followed by 60–120 minutes incubation at 37°C to allow the Cot-1 DNA to block repetitive sequences. The mixture was hybridized on an array containing approximately 5000 clones spotted in triplicate and spread along the whole genome with an average resolution of 1.0 Mb. The clones are comprised of the Sanger BAC clone set with an average resolution along the whole genome of 1.0 Mb [40], the OncoBac set [41], and selected clones of interest, obtained from the Children's Hospital Oakland Research Institute (CHORI). The selected clones comprise a collection of BAC clones on chromosome 6 filling the gaps larger than 1 Mb, and full-coverage contigs on specific regions on chromosomes 8, 13 and 20. Hybridization was performed in a in a hybridization station (Hybstation12 – Perkin Elmer Life Sciences, Zaventem, BE) and incubated for 38 h at 37°C. After hybridization, slides were washed in a solution containing 50% formamide, 2× SCC, pH 7 for 3 minutes at 45°C, followed by 1 minute wash steps at room temperature with PN buffer (PN: 0.1 M sodiumphosphate, 0.1% nonidet P40, pH 8), 0.2× SSC, 0.1× SCC and 0.01× SCC.

### Image acquisition and data analysis

Images of the arrays were acquired by scanning (Agilent DNA Microarray scanner- Agilent technologies, Palo Alto, USA) and quantification of the signal and background intensities for each spot for the two channels Cy3 and Cy5 was performed by Imagene 5.6 software (Biodiscovery Ltd, Marina del Rey, CA, USA). Local background was subtracted from the signal median intensities and tumours to reference ratios were calculated. The ratios were normalized against the mode of the ratios of all autosomes. Clones with poor quality of one of the triplicates and hybridization with a standard deviation (SD) ≤ 0.22 and clones with > 50% missing values in all adenomas were excluded, leaving 4648 clones for further analysis. All subsequent analyses were done considering the clone position from the UCSC May2004 freeze of the Human Golden Path.

Array CGH smooth [42,43], was used for automated detection of breakpoints to determine copy number gains and losses. Since variation in quality is observed in DNA obtained from formalin-fixed paraffin-embedded gastric tissues, different smoothing parameters were applied, depending on the quality of the hybridization. For array CGH profiles with a standard deviation smaller or equal to 0.15, between 0.15 and 0.20 or between 0.20 and 0.22, the applied smoothing parameters to determine gains and losses were 0.10, 0.15 and 0.20 respectively. Log_2 _tumour to reference ratio above 1 was regarded as amplification.

### Statistical analysis

Unsupervised hierarchical cluster analysis was performed to analyze the distributions of the genomic profiles of all adenomas using TMEV software 3.0.3 [44]. Based on normalized smoothed log_2 _tumour to normal fluorescence intensity ratios, a hierarchical tree was constructed using the parameters complete linkage and euclidean distance. Pearson Chi-square test was used for analyzing correlations between cluster membership and adenoma type (SPSS 11.5.0 for windows, SPSS Inc, Chicago, IL, USA). P-values less than 0.05 were considered to be significant.

Supervised analysis was used for identifying chromosomal regions specific for the two adenoma types using CGH Multiarray and CGH Multiarray Region [45,46]. Based on normalized smoothed log_2 _tumour to normal fluorescence intensity ratios, p-values were calculated for the significance of difference of values for each clone between pyloric gland and intestinal-type adenomas, using a Wilcoxon test with ties. To correct for multiple testing, a permutation-based false discovery rate (FDR) was calculated [47].

## Competing interests

The author(s) declares that there are no competing interests.

## Authors' contributions

TB performed all the data analysis and wrote the manuscript. BC helped with data analysis and writing of the manuscript and helped in coordinating the study. TM performed the DNA isolations and TM and RR performed the array CGH experiments. CM and PS helped with the DNA isolations. NG revised the adenomas derived from Germany. MV and MS provided the material obtained from Germany. CV was involved in reviewing the manuscript and supervision of the study. ES and AM were involved in development and establishing the BAC arrays. BY was involved in development of the BAC arrays and provided the facilities for the microarray experiments. FC provided the material obtained form Portugal, conceived the study and was involved in critically reviewing the manuscript. GM revised the material obtained from Portugal, coordinated the study and helped to draft the manuscript. All authors read and approved the manuscript.
